# An Improved Dengue Virus Serotype-Specific Non-Structural Protein 1 Capture Immunochromatography Method with Reduced Sample Volume

**DOI:** 10.3390/bios15120802

**Published:** 2025-12-07

**Authors:** Warisara Sretapunya, Thitiya Buranachat, Montita Prasomthong, Rittichai Tantikorn, Areerat Sa-ngarsang, Sirirat Naemkhunthot, Laddawan Meephaendee, Pattara Wongjaroen, Chika Tanaka, Yoriko Shimadzu, Katsuya Ogata, Kunihiro Kaihatsu, Ryo Morita, Michinori Shirano, Juthamas Phadungsombat, Tadahiro Sasaki, Ritsuko Kubota-Koketsu, Yoshihiro Samune, Emi E. Nakayama, Tatsuo Shioda

**Affiliations:** 1 Nakhon Nayok Hospital, Nakhon Nayok 26000, Thailand; warisara_toon@hotmail.com (W.S.); thitiyaboo@gmail.com (T.B.); maxshineesnw@gmail.com (M.P.); rittichai2509@gmail.com (R.T.); 2 National Institute of Health, Department of Medical Science, Ministry of Public Health, Nonthaburi 11000, Thailand; areerat.sangarsang@gmail.com (A.S.-n.); sirirat.n@dmsc.mail.go.th (S.N.); laddawan.m@dmsc.mail.go.th (L.M.); pattara.w@dmsc.mail.go.th (P.W.); 3 VisGene Ltd., Osaka 562-0033, Japan; chika@visgene.com (C.T.); shimazu@visgene.com (Y.S.); ogata-k@visgene.com (K.O.); kaihatsu@visgene.com (K.K.); 4 Department of Infectious Diseases, Osaka City General Hospital, Osaka 534-0021, Japan; ryo10071007mrt@yahoo.co.jp (R.M.); shirano@wonder.ocn.ne.jp (M.S.); 5 Department of Infectious Diseases, Osaka City Juso Hospital, Osaka 532-0034, Japan; 6 Department of Viral Infections, Research Institute for Microbial Diseases, The University of Osaka, Osaka 565-0871, Japan; juthamas@biken.osaka-u.ac.jp (J.P.); sasatada@biken.osaka-u.ac.jp (T.S.); koketsu@biken.osaka-u.ac.jp (R.K.-K.); u521240h@alumni.osaka-u.ac.jp (Y.S.); emien@biken.osaka-u.ac.jp (E.E.N.)

**Keywords:** dengue virus, serotype discrimination, rapid test, immunochromatography, NS1

## Abstract

The four serotypes of dengue virus (DENV), types 1 to 4 (DENV-1 to DENV-4), exhibit approximately 60% identity in the encoded amino acid residues of viral proteins. Reverse transcription of RNA extracted from patient serum specimens followed by PCR amplification with serotype-specific probes is the current standard technique for DENV serotyping. However, this method is time- and cost-consuming, and rapid detection systems with low cost are desirable. Previously, we developed a prototype serotype-specific immunochromatography system. That system was composed of four strips with four corresponding distinct sample buffers, each specifically detecting a single DENV serotype. In the present study, we improved this system by combining pairs of strips into one lateral-flow cassette each, providing DENV-1 and DENV-2 detection in one device and DENV-3 and DENV-4 detection in a second device; this strategy successfully reduced the required sample volume. Furthermore, we were able to adjust the composition of the sample buffers such that a single sample buffer sufficed for all four DENV serotype detection reactions, allowing much easier handling of the devices. Evaluation of this new device against laboratory and clinical DENV isolates and clinical specimens from DENV-infected individuals showed sensitivity that was comparable to that of our previous version, yielding serotype specificity of 100%. These new devices are expected to be of use in the clinical setting, accelerating both prospective and retrospective epidemiological studies.

## 1. Introduction

Dengue is one of the most important arthropod-borne diseases in tropical and subtropical areas. Dengue is caused by dengue virus (DENV) infection and is transmitted by *Aedes aegypti* mosquitos and other species in the *Aedes* genus [1]. The number of reported dengue cases has been increasing in recent years, with approximately a half-million cases in 2000 and 14.6 million cases in 2024. It is estimated that 100–400 million infections occur annually and that half of the world population is currently at risk of dengue infection [2].

DENV belongs to the genus *Orthoflavivirus*, family *Flaviviridae* [3]. The genome of this virus consists of a single-stranded, positively oriented RNA approximately 11 kb in length, and encodes three structural (capsid, precursor-membrane, and envelope) and seven non-structural (NS1, NS2A, NS2B, NS3, NS4A, NS4B, and NS5) proteins in order from its 5’ end. Structural proteins form the virus particles; non-structural proteins facilitate replication and packaging of the viral genome, processing of viral polyproteins, and evasion of host defense systems [4]. DENV consists of four antigenically distinct serotypes, DENV-1, DENV-2, DENV-3, and DENV-4. These four serotypes of DENV exhibit approximately 60% identity in the encoded amino acid residues of viral proteins; each serotype is further divided into several genotypes. The major circulating serotype, genotype, and even the clade/lineage within a given genotype are known to change frequently over time in each endemic area [5,6,7,8,9,10,11,12,13,14].

DENV infection presents with a wide range of effects, ranging from asymptomatic infection or mild fever to much more severe fatal shock syndrome due to plasma leakage, with the fatality typically manifesting after the fever has resolved. Patients who are infected with DENV for a second time exhibit a higher risk for severe dengue compared with those infected for the first time [2]. Symptomatic secondary DENV infections are typically the result of infection by virus of a serotype differing from that of the primary infection, since homotypic anti-viral immunity persists following the initial infection. As assessed in a Nicaraguan cohort, pre-existing suboptimal levels of anti-DENV immunity, rather than the absence of any anti-DENV immunity, is associated with severe dengue [15]. Therefore, it would be beneficial to know the serotypes of infecting DENV to permit more-precise determination of an individual’s risk for severe disease. Furthermore, we observed that the proportion of patients with severe dengue doubled upon a huge sudden outbreak of DENV-3 in 2019 in Bangladesh [8]. Again, it would be beneficial to know the infecting DENV serotype to assess the risks for severe dengue in a given population.

Reverse transcriptase–polymerase chain reaction (RT-PCR) followed by sequencing of the DENV genome from patient blood remains the most-reliable method for serotyping clinical samples. However, this method is time-consuming and costly and is not suitable for clinical settings and epidemiological studies with large numbers of specimens. In previous work, we reported the development of a prototype DENV serotype-specific non-structural protein 1 (NS1) capture immunochromatography (IC) kit [16]; we employed this approach because NS1 is already used as a target for antigen capture rapid diagnostic tests [17,18,19,20,21,22]. Our kit was composed of four strips, each providing detection for a single DENV serotype, and was able to differentiate the four DENV serotypes in laboratory and clinical isolates as well as in clinical specimens. However, our assay required a minimal sample volume of 120 μL; furthermore, the handling of four different strips with four different sample buffers was labor intensive and so was expected to hamper widespread adoption of this kit. In the present study, we optimized our kit by combining pairs of strips into single lateral flow cassettes. Here, we show the improved utility of our updated DENV serotyping kit based on the evaluation of preclinical and clinical samples.

## 2. Materials and Methods

### 2.1. Ethics Statement

Protocols on the use of clinical specimens at the Nakhon Nayok Hospital were reviewed and approved by the Human Research Ethics Committee (REC) of the Nakhon Nayok Hospital (Nakhon Nayok Hospital REC No. 11/2020). Protocols on the use of clinical specimens at the Research Institute for Microbial Diseases, Osaka University, were reviewed and approved by the Institutional Review Board of the Research Institute for Microbial Diseases, Osaka University (Approval No. 2023-15).

### 2.2. Immunochromatographic Assay Preparation

The combinations of the anti-DENV NS1 mouse monoclonal antibodies used in the present study are summarized in Table 1. Detailed characteristics of these monoclonal antibodies (A, B, D, E, F, G, and H) have been described previously [11]. Use of these antibodies was approved by ARKRAY, Inc. (Kyoto, Japan). Test lines were generated by immobilizing capture antibodies onto a nitrocellulose membrane at 0.125 μg/test. To prepare the control line, anti-mouse immunoglobulin G (IgG; Immuno Probe Co., Ltd., Saitama, Japan) was immobilized onto a nitrocellulose membrane at 0.125 μg/test to capture mouse IgG. The detection antibodies were labeled with colloidal gold, impregnated onto glass fibers, dried, and placed at the conjugate pad between the test line and the sample-soaking region. The nitrocellulose membrane and glass fiber pad were assembled with a glass fiber sample pad on a plastic sheet. The assembled device was stored in a plastic cassette.

### 2.3. Viruses

DENV-1 (Mochizuki strain), DENV-2 (Strain 16681), DENV-3 (Strain H87), DENV-4 (Strain H241), and six clinical isolates obtained from Thai patients with dengue (Supplemental Table S1) were propagated in C6/36 cells, and the virus titer in each culture supernatant was determined using real-time PCR [23].

### 2.4. Sequence Determination of DENV Strains

Nucleotide sequences of the DENV strains were determined as described previously [5].

### 2.5. Clinical Specimens in Thailand

Serum samples were collected from 142 patients who presented at the Nakhon Nayok Hospital during 2020–2021 and were suspected to be infected with DENV. Viral RNA was extracted from 100 μL of clinical serum specimens using a QIAamp Viral RNA Mini Kit (Qiagen, Hilden, Germany) according to the manufacturer’s instructions. The DENV serotyping and chikungunya virus (CHIKV) data as well as each viral load were obtained by Multiplex Real-Time PCR using an abTESTM DEN/CHIKU 5 qPCR II Kit (AITbiotech Pte, Ltd., The Rutherford Science Park 1, Singapore). An SD-Bioline brand NS1 antigen and anti-DENV IgG and IgM detection devices (Alere Medical/Abbott, Waltham, MA, USA) and an ichroma™ immunoassay (Boditech Med, Inc., Chuncheon-si, Korea) were used according to the respective manufacturer’s instructions.

### 2.6. Clinical Specimens in Patients in Japan

Ten febrile patients (returning from tropical or subtropical foreign countries to Japan) presenting at Osaka City Juso Hospital or Osaka City General Hospital during 2022–2024 who were diagnosed (using the SD-Bioline NS1 antigen and anti-DENV IgG and IgM detection devices (Alere Medical/Abbott, Waltham, MA, USA)) to be harboring DENV infections were recruited for the present study. Viral RNA was extracted from 140 μL of clinical serum or plasma specimens using a QIAamp viral RNA mini kit according to the manufacturer’s instructions. The viral load data were quantified using a one-step SYBR Green I–based quantitative RT-PCR reaction specific for DENV, employing previously described primer pairs and protocols [24]. DENV-positive RNA samples then were analyzed according to the manufacturer’s instructions using a commercial dengue subtyping multiplex kit (Primerdesign Ltd., Manchester, UK) for DENV serotype determination.

### 2.7. Antigen Detection Using the IC Devices

An aliquot (30 μL) of virus stock subjected to serial dilution in Minimal Essential Medium (MEM) or DENV patient serum was mixed with 30 μL of the specific buffer. The resulting mixture was dispensed into the sample hole of the device, which then was incubated for 15 min at room temperature. Appearance of signals at both the test and control lines was regarded as a positive reaction, while appearance of a signal at only the control line was regarded as a negative reaction. For quantitative evaluation using laboratory and clinical strains of DENV, the test band intensities were measured using a chromatogram reader (Hamamatsu Photonics, Bridgewater, NJ, USA) and expressed as the attenuation of reflected brightness in milli-absorbance units (mAbs). Bands with an intensity > 15 mAbs were visible by eye.

## 3. Results

### 3.1. Reconstruction of a DENV Serotype-Specific NS1 Capture IC Kit

**Figure 1:**
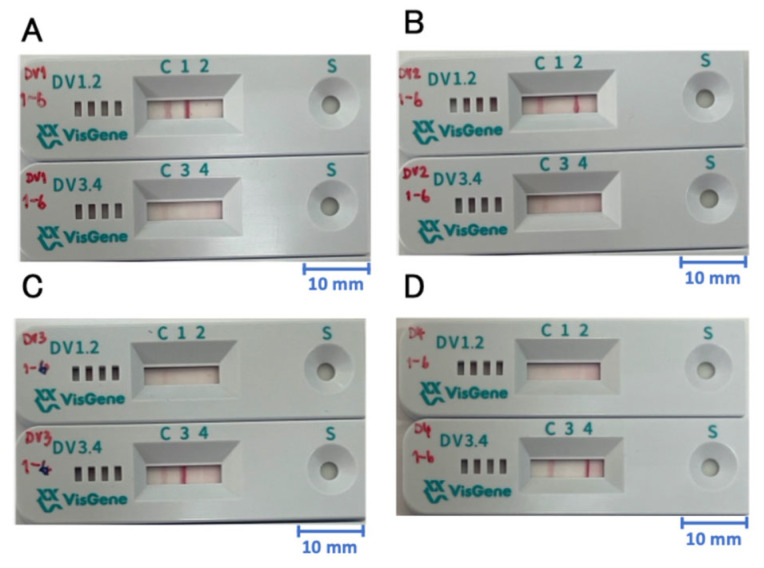
Dengue virus serotyping NS1 capture immunochromatography. In the DV1.2 device, C, 1, and 2 denote positions of control line, dengue virus type 1 test line, and dengue virus type 2 line, respectively. In the DV3.4 device, C, 3, and 4 denote positions of control line, dengue virus type 3 test line, and dengue virus type 4 test line, respectively. S denotes the sample hole, Positive results for dengue virus known to be type 1 (**A**), type 2 (**B**), type 3 (**C**), and type 4 (**D**) are shown. A scale of 10 mm is shown in each panel.

Our previous DENV serotype-specific NS1 capture IC kit consisted of four IC strips, each detecting one serotype of DENV [16]. Thirty microliters of specimen was required for each strip, meaning that a total specimen volume of 120 μL was required for serotyping. In the present study, to reduce the required sample volume and to improve the utility of the kit, we combined these four strips pair-wise into two lateral flow IC strips contained in plastic cassettes. Table 1 shows the combinations of monoclonal antibodies used in the new strips. One lateral flow IC strip cassette was designed to detect either DENV-1 or DENV-2 at two distinct test lines (Figure 1A,B). Similarly, another cassette was designed to detect either DENV-3 or DENV-4 at two distinct test lines (Figure 1C,D).

### 3.2. Detection of NS1 in Cultured Viruses

**Figure 2:**
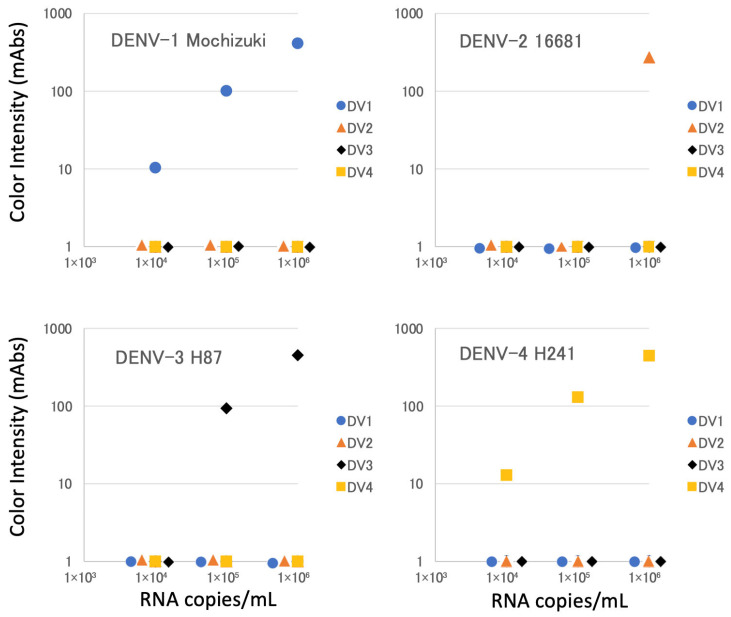
Evaluation of serotype specificity using laboratory strains. DV1, DV2, DV3, and DV4 denote results on test lines for dengue virus type 1, type 2, type 3, and type 4, respectively. On the horizontal axes, the overlapping symbols are arranged side by side.

We first evaluated the serotype specificity of our new devices (each consisting of a cassette) by using cultured laboratory strains of DENV of known serotypes. As shown in Figure 1 and Figure 2, the Mochizuki DENV-1 strain and the 16681 DENV-2 strain were specifically detected by DENV-1-specific and DENV-2-specific test lines, respectively, in the DENV-1 and 2-specific device (DV1.2). Similarly, the H87 DENV-3 strain and the H241 DENV-4 strain were specifically detected by DENV-3-specific and DENV-4-specific test lines, respectively, in the DENV-3 and 4-specific device (DV3.4). There was no cross-reaction among these four strains, even at the highest input concentration of 10^6^ copies/mL (Figure 1 and Figure 2).

**Figure 3:**
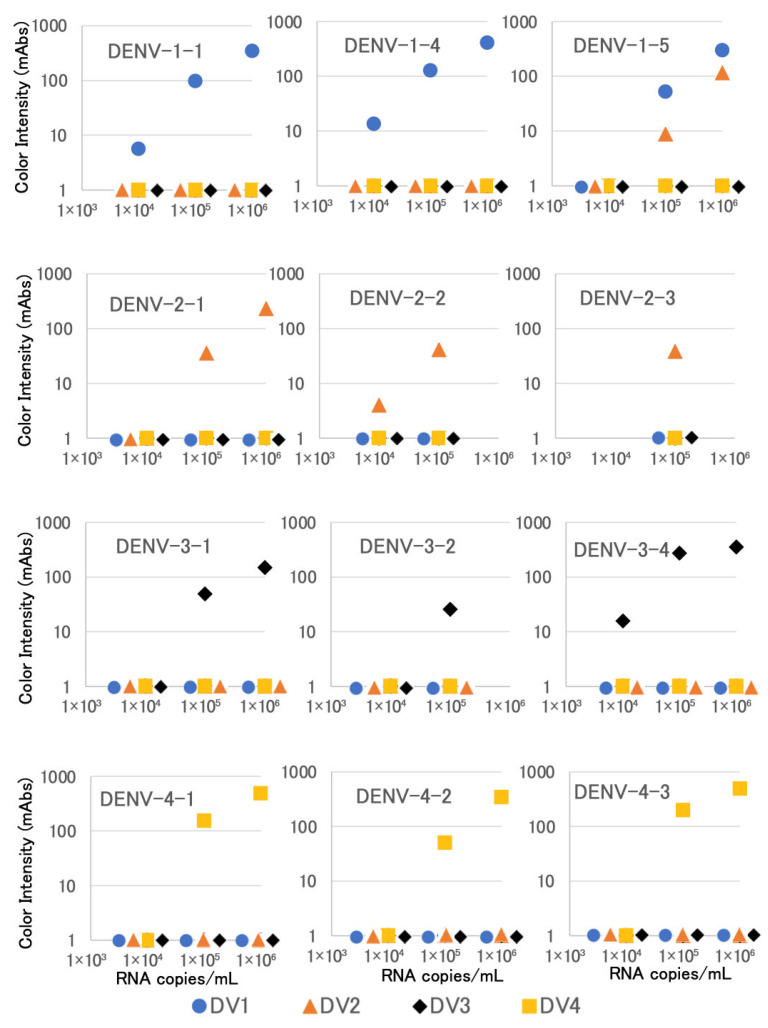
Evaluation of serotype specificity using clinical isolates. DV1, DV2, DV3, and DV4 denote results on test lines for dengue virus type 1, type 2, type 3, and type 4, respectively. On the horizontal axes, the overlapping symbols are arranged side by side.

We next evaluated our new device by using recent isolates of DENV. For each serotype, we tested three distinct clinical isolates and found that all of the 12 tested DENV isolates were correctly serotyped at input concentrations of at least 10^5^ copies/mL (Figure 3). Some clinical isolates, such as DENV-1-1, DENV-1-4, DENV-2-2, and DENV-3-4, were correctly serotyped at an input concentration of 10^4^ copies/mL. One DENV-1 isolate, DENV-1-5, which was not used in our evaluation of the previous version of serotyping kit [16], showed cross-reactivity at the DENV-2 test line, but the intensities of the DENV-1 test lines were higher than those at the DENV-2 test lines at input concentrations of 10^5^ and 10^6^ copies/mL (Figure 3). Amino acid sequence alignment of the NS1 proteins of DENV-1 and DENV-2 strains (Table S1) showed that DENV-1-5 encoded the protein with tyrosine and asparagine at the 323rd and 347th positions, respectively, where all the other DENV-1 strains encoded NS1 with phenylalanine and lysine at these positions. In DENV-2 strains, these positions were encoded as tyrosine and asparagine, as seen with DENV-1-5 (Table S1). Therefore, it is possible that the epitope of the antibodies used in the DENV-1- and -2-specific device corresponds to the C-terminus of NS1, and that amino acid variations at positions 323 and/or 347 affect cross-reactions in this device.

### 3.3. Detection of NS1 in Clinical Specimens in Thailand

We next performed a clinical trial of the new devices at the Nakhon Nayok Hospital, Thailand. We used sera which were confirmed (by RT-PCR) to represent 49 DENV-1-, four DENV-2-, one DENV-3-, and one CHIKV-positive cases, as well as 87 cases that tested (by RT-PCR) negative for these viruses. Therefore, our clinical dataset was heavily skewed toward DENV-1. As shown in Table 2, our new devices detected DENV-1 NS1 accurately in 31 of 49 DENV-1 cases, corresponding to a sensitivity of 63.27% with the 95% confidential interval (CI) of 49.77–76.76. The mean Ct value of the viral load in these 31 positive cases was 23.2 with a standard deviation (SD) of 5.7, while that of the 18 negative cases was 30.1 with a SD of 5.1. This difference was statistically significant (*p* = 0.00012, *t*-test). These results indicated that specimens with lower viral loads tend to show negative results. The DENV-1 test lines of our device did not show any cross-reaction with DENV-2, DENV-3, or CHIKV, nor with RT-PCR-negative cases, corresponding to a specificity of 100% (Table 2).

All of the specimens also were assessed (in parallel) for the presence of NS1 protein, anti-DENV IgM, and anti-DENV IgG as determined by two commercially available kits. The Bioline™ Dengue Duo kit detected NS1 in an additional five specimens among the 18 negative cases, but failed to detect NS1 in two specimens among the 31 cases that tested positive (showing a DENV-1 test line) using our device. The ichroma™ NS1 kit detected the protein in six specimens among the 18 cases that tested negative using our device, but the ichroma™ kit also failed to detect three specimens among the 31 cases that tested positive (showing a DENV-1 test line) using our device. Therefore, the sensitivity of the Bioline™ Dengue Duo kit and the ichroma™ NS1 kit against these 49 DENV-1 specimens was 69.39% with the 95%CI of 56.48–82.29, which was almost comparable to the sensitivity (63.27%) obtained with our revised device (*p* = 0.6694, Fisher’s exact test). Among the five specimens that tested positive by the Bioline™ Dengue Duo kit and negative by the DENV-1 test line of our device, three tested positive by the ichroma™ NS1 kit. There were two specimens that tested positive by the DENV-1 test line of our device and negative by the Bioline™ Dengue Duo kit. Another three specimens showed positive by the DENV-1 test line of our device and negative by ichroma™ NS1 kit. These results suggested that the observed small differences in kit sensitivity reflect differences in the epitope coverage spectra of the antibodies used in the individual kits, rather than the reduced number of epitopes covered by antibodies used in our DENV serotyping kit compared with the Bioline™ and ichroma™ kits. It is possible that our new device may show higher sensitivity than those commercially available DENV detection kits in other sample set. In fact, our prototype DENV serotyping kit showed higher sensitivity than the commercially available DENV detection kit [16].

Previous studies reported that the presence of anti-DENV antibodies within specimens affected the sensitivities of DENV NS1 detection kits [25]. Indeed, of the 18 negative cases that tested negative by the DENV-1 test line of our device, two (10.53%) contained detectable levels of both anti-DENV IgM and IgG, and 11 (61.11%) contained detectable levels of anti-DENV IgG alone by one or both of the Bioline™ Dengue Duo kit and the ichroma™ NS1 kit. On the other hand, of the 31 cases that tested positive by the DENV-1 test line of our device, two (6.45%) contained detectable levels of both anti-DENV IgM and IgG, and seven (22.58%) contained detectable levels of anti-DENV IgM alone by one or both of the Bioline™ Dengue Duo kit and the ichroma™ NS1 kit. The difference was statistically significant (*p* = 0.0066, Fisher’s exact test). Therefore, the results of the present study confirm the notion that the presence of antibodies against DENV NS1 in patient samples interferes with NS1 detection by rapid test devices.

As shown in Table 3, our device detected DENV-2 NS1 accurately in two of four DENV-2 cases. As seen with DENV-1 cases, the mean Ct value of viral load in these two positive cases was lower (with a mean value of 19.9) than that of the two negative cases (mean value of 27.8). Also, the DENV-2 test lines of our device did not show any cross-reaction with DENV-1, DENV-3, or CHIKV, nor with RT-PCR-negative cases (Table 3). The Bioline™ Dengue Duo kit showed the same results as our device. The ichroma™ NS1 kit detected one more specimen which both our device and the Bioline™ Dengue Duo kit failed to detect the NS1. In an single DENV 2 specimen which all three kits failed to detect the NS1, the Bioline™ Dengue Duo kit detected anti-DENV IgM, while the ichroma™ NS1 kit detected anti-DENV IgG, also confirming the notion that the presence of anti-DENV antibodies interferes with NS1 detection.

The present study included only one DENV-3 case, and our device accurately detected DENV-3 NS1 in this specimen (Table 4). On the other hand, no DENV-4 clinical cases were available for analysis by our device. However, the DENV-3 and DENV-4 test lines of our device did not show any cross-reactions with other DENV serotypes, with CHIKV, or with RT-PCR-negative cases (Table 4 and Table 5). The Ct value of the DENV-3 specimen was 34.5, even higher than those of most of the negative cases of our DENV-1 and DENV-2 test lines. This case lacked anti-DENV antibodies. It is likely that the lack of anti-DENV antibodies allowed NS1 detection even in the low viral load.

### 3.4. Detection of NS1 in Clinical Specimens in Japan

Since the composition of the Thailand specimens used in the present study was highly biased to DENV-1, we added a small-scale clinical trial in Japan to our study. We used serum or plasma specimens from 10 dengue cases in which infection was confirmed by RT-PCR; all represented travelers who were returning from tropical or subtropical areas. These 10 cases included three DENV-1 specimens, five DENV-2 specimens, one DENV-3 specimen, and one DENV-4 specimen. As shown in Tables S2–S5, our devices accurately detected NS1 of the respective serotype of DENV, except for a single DENV-2 case shown to have a low viral load (13.9 copies/mL). As seen for the Thailand specimens, our devices did not show any cross-reactivity with other serotypes of DENV, resulting in 100% specificity (Tables S2–S5).

## 4. Discussion

In the present study, we were able to modify our prototype DENV serotyping device [16] for use with reduced sample volumes and simplified procedures. As seen with the prototype device, the modified devices successfully differentiated the four serotypes of DENV in laboratory and clinical virus isolates (Figure 2 and Figure 3) and in clinical specimens collected in Thailand (Table 2, Table 3, Table 4 and Table 5) and Japan (Tables S2–S5). The required sample volume for the revised kit is now 60 μL, half of that needed by our prototype kit. Furthermore, combining four strips into two and using a single buffer rather than four different buffers reduces frequency of kit handing mistakes. The kit usability is now as easy as the existing simple commercially available DENV detection kits. Therefore, our revised devices not only permitted easier and simpler handling in actual clinical settings but also are expected to expand the application of the kit by facilitating retrospective epidemiological studies on precious preserved specimens with small sample volumes. It should also be noted that our revised devices passed a stability testing at 55 °C for 3 months for the use in tropical and subtropical filed condition.

As described above, most of the Thailand clinical specimens were DENV-1. We therefore added another clinical trial in Japan using dengue-positive specimens obtained from travelers returning from tropical and subtropical areas. When we combined the results from Thailand and Japan, the study population totaled 52 DENV-1 cases, nine DENV-2 cases, two DENV-3 cases, and one DENV-4 case. Among these 64 cases total, our new device successfully detected NS1 in 34 DENV-1 cases, six DENV-2 cases, two DENV-3 cases, and one DENV-4 case, corresponding to sensitivities of 65.38%, 66.67%, 100%, and 100% for DENV-1 through -4 cases (respectively). Given that no cross-reactions were detected with other serotypes of DENV, with CHIKV, or with RT-PCR-negative cases, the specificity of the revised devices was calculated as 100% for all four DENV test lines and the overall agreements for DENV-1 to -4 were 88.16%, 98.03%, 100%, and 100%, respectively. Although the clinical trials of the present study covered all four serotypes of DENV, the numbers of DENV-2, DENV-3, and DENV-4 cases were still limited. Therefore, a further clinical trial with a larger number of these DENV serotypes is needed to validate our revised devices.

Of the 49 DENV-1 clinical specimens obtained in Thailand, our new device detected this serotype with 63.27% sensitivity (Table 2); for comparison, two commercially available DENV NS1 detection kits, the Bioline™ Dengue Duo kit and the ichroma™ NS1 kit, exhibited a slightly higher sensitivity of 69.39%. However, as noted above, the Bioline™ Dengue Duo kit and the ichroma™ NS1 kit failed to detect DENV NS1 in two and three specimens, respectively, in which our new device successfully detected NS1. It is thus likely that the different antibodies used in each of these kits detect distinct spectra of NS1 epitopes in clinical specimens, such that kit sensitivities may vary in clinical trials performed in different geographical areas where different genotypes and lineages of the DENV serotypes circulate [9,25,26,27,28,29]. It is also theoretically possible that a reduction in sample volume from 120 µL to 60 µL might decrease analytical sensitivity. However, our evaluation demonstrated that the limit of detection was essentially unchanged, and no significant loss in signal intensity was observed. This result is likely due to optimization of other parts of the device, such as sample pad treatment and conjugate release efficiency.

It was evident that two major factors reducing the kit sensitivity were low DENV load and the presence of anti-DENV antibodies within the specimens. It is reasonable to speculate that anti-NS1 antibodies within clinical specimens would mask the NS1 epitopes and reduce the kit sensitivity. The different kinetics of DENV RNA from that of the NS1 reported by several articles [30,31,32,33] may also affect the kit sensitivity. The sensitivity of our device would thus vary depending on the sampling days after the onset of symptoms in patients and the proportion of specimens with anti-DENV NS1 antibodies. It would be beneficial to combine our device with anti-DENV IgM and IgG detection unit to detect DENV infections in specimens with broader range of sampling days after the onset of dengue symptoms.

Regarding specificity, our new devices did not show any cross-reactions with other serotypes of DENV or CHIKV, nor with RT-PCR-negative cases, in clinical trials. However, there was a single DENV-1 isolate that also reacted weakly with the DENV-2 test line of our respective device (Figure 3). While we ruled out the possible presence of a DENV-2 sequence in the DENV-1-containing specimen by performing RT-PCR followed by sequence determination on the viral genome, we did note that the NS1 protein encoded by this DENV-1 isolate included two DENV-2-like amino acid residues at the 323rd and 347th positions (Table S1); we infer that one or both of these residues contribute to the cross-reaction observed for this specimen in the present study. It is possible that the genotypes of DENVs other than those circulating in Thailand encode slightly different NS1 sequences, and hence, show different reactivities in our devices. Therefore, our present result confirms the necessity of conducting clinical trials of our devices (and indeed, any such analytical tools) in geographical regions other than Thailand, where DENVs encoding slightly different NS1 sequences may circulate.

In the 3D structure of DENV-1 NS1 [34], the position 323 is buried within the NS1 molecule, while the position 347 is exposed on the NS1 molecular surface as shown in Figure S1. In fact, the position 347 was reported to be within one of the discontinuous binding sites of murine anti-NS1 monoclonal antibody 2B7. Furthermore, the 2B7 bound strongly to DENV-1 but only weakly to DENV-2 [34]. Therefore, it is tempting to speculate that introduction of DENV-2-like amino acid at the 347th position of DENV-1 NS1 changed the surface epitope structure and allowed the DENV-2 specific antibodies in our device to moderately bind to the DENV-1-5 clinical isolate. Further studies including mutagenesis at positions 323 and 347 of DENV-1 NS1 are needed to confirm this speculation.

The limitations of the present study include small number of clinical specimens with skewed distribution of DENV serotypes. The most samples originated in a single country, Thailand, which is another limitation of our study, since there are divergent DENV strains in different geographical regions as described above. To further validate our new DENV serotyping device, it is absolutely essential to conduct additional studies using more specimens originating from multiple geographic regions within dengue-endemic areas. It is worth noting here that our new DENV serotyping device yielded a sensitivity of 82.05% (95% CI 73.53–90.57) in Bangladesh, a south Asian country, where our new DENV serotyping device was able to correctly detect 41 DENV-2, 21 DENV-3, and one DENV-4 among 78 DENV confirmed patients [35].

In conclusion, we successfully modified our prototype DENV serotyping kit to generate a revised version with reduced sample volume requirements and simplified procedures. Our new kit can be used in the clinical setting and in the context of both prospective and retrospective epidemiological studies. Nonetheless, additional studies will be needed to further generalize our conclusions.

## Figures and Tables

**Table 1:** Antibodies used in the immunochromatographic devices.

Device	Detection Antibody		Capture Antibodies	
	(gold-label)		(membrane-bound)	
DENV-1 and -2 specific	A		E	F
DENV-3 and -4 specific	B	D	G	H

**Table 2:** Serotyping results at the DENV-1 test line for Thailand clinical specimens.

DENV-1	DENV-1 IC Results		Sensitivity (%)	Specificity (%)	OAA (%) *
PCR Results	+	−	95% CI **	95% CI **	95% CI **
+	31	18	63.27	100	87.32
−	0	93	49.77–76.76	100–100	81.85–92.80

* Overall agreement. ** Confidence interval.

**Table 3:** Serotyping Results at DENV-2 test line of Thailand clinical specimens.

DENV-2	DENV-2 IC Results		Sensitivity (%)	Specificity (%)	OAA (%) *
PCR Results	+	−	95% CI **	95% CI **	95% CI **
+	2	2	50	100	98.59
−	0	138	1–99	100–100	96.645–100

* Overall agreement. ** Confidence interval.

**Table 4:** Serotyping Results at DENV-3 test line of Thailand clinical specimens.

DENV-3	DENV-3 IC Results		Sensitivity (%)	Specificity (%)	OAA (%) *
PCR Results	+	−	95% CI **	95% CI **	95% CI **
+	1	0	100	100	100
−	0	141	−	100–100	100–100

* Overall agreement. ** Confidence interval.

**Table 5:** Serotyping Results at DENV-4 test line of Thailand clinical specimens.

DENV-4	DENV-4 IC Results		Sensitivity (%)	Specificity (%)	OAA (%) *
PCR Results	+	−	95% CI **	95% CI **	95% CI **
+	0	0	−	100	−
−	0	142	−	100–100	−

* Overall agreement. ** Confidence interval.

## Data Availability

The original contributions presented in this study are included in the article/Supplementary Materials. Further inquiries can be directed to the corresponding author.

## Supplementary Materials

The following are available online at https://www.mdpi.com/article/10.3390/bios15120802/s1, Table S1: NS1 protein amino acid sequences; Table S2: Serotyping results for DENV-1 test line of clinical specimens in Japan; Table S3: Serotyping results for DENV-2 test line of clinical specimens in Japan; Table S4: Serotyping results for DENV-3 test line of clinical specimens in Japan; Table S5: Serotyping results for DENV-4 test line of clinical specimens in Japan; Figure S1: The 3D structure of dengue virus type 1 NS1 protein tetramer (PDB ID:6WEQ). (A) Spheres indicate either phenylalanine (F) residue at the 323rd or lysine (K) residue at the 347th positions. (B) Lysine residues (K) at the 347th positions are exposed on the molecular surface, while phenylalanine (F) residues at the 323rd positions are buried within the molecule.
