# Supplementary material for: An Improved Dengue Virus Serotype-Specific Non-Structural Protein 1 Capture Immunochromatography Method with Reduced Sample Volume
Source: Biosensors (Basel). 2025 Dec 7;15(12):802. doi: 10.3390/bios15120802 (PMC12730692; doi:10.3390/bios15120802)
Supplement: Supplementary file 1 [file biosensors-15-00802-s001.zip › Tables S2-S5.pdf]

Table S2. Serotyping Results at DENV-1 test line of clinical specimens in Japan.

DENV-1<br>PCR resulls	DENV-1 IC Results<br>+	 -	Sensitivity(%)<br>95% CI**	Specificity (%)<br>95% CI**	OAA (%)*<br>95% CI**
+	3	0	100	100	100
-	0	7	100-100	100-100	100-100

*Overall agreement. **Confidence interval

Table S3. Serotyping Results at DENV-2 test line of clinical specimens in Japan.

DENV-2<br>PCR results	DENV-2 IC Results<br>+	 -	Sensitivity(%)<br>95% CI**	Specificity (%)<br>95% CI**	OAA (%)*<br>95% CI**
+	4	1	80	100	90
-	0	5	44.94-100	100-100	71.41-100

*Overall agreement. **Confidence interval

Table S4. Serotyping Results at DENV-3 test line of clinical specimens in Japan.

DENV-3<br>PCR resulls	DENV-3 IC Results<br>+	 -	Sensitivity(%)<br>95% CI**	Specificity (%)<br>95% CI**	OAA (%)*<br>95% CI**
+	1	0	100	100	100
-	0	9	-	100-100	100-100

*Overall agreement. **Confidence interval

Table S5. Serotyping Results at DENV-4 test line of cnical specimens in Japan.

DENV-4<br>PCR results	DENV-4 IC Results<br>+	 -	Sensitivity(%)<br>95% CI**	Specificity (%)<br>95% CI**	OAA (%)*<br>95% CI**
+	1	0	100	100	100
-	0	9	-	100-100	100-100

*Overall agreement. **Confidence interval
