# Supplementary figures and images for: An Improved Dengue Virus Serotype-Specific Non-Structural Protein 1 Capture Immunochromatography Method with Reduced Sample Volume
Source: Biosensors (Basel). 2025 Dec 7;15(12):802. doi: 10.3390/bios15120802 (PMC12730692; doi:10.3390/bios15120802)

A

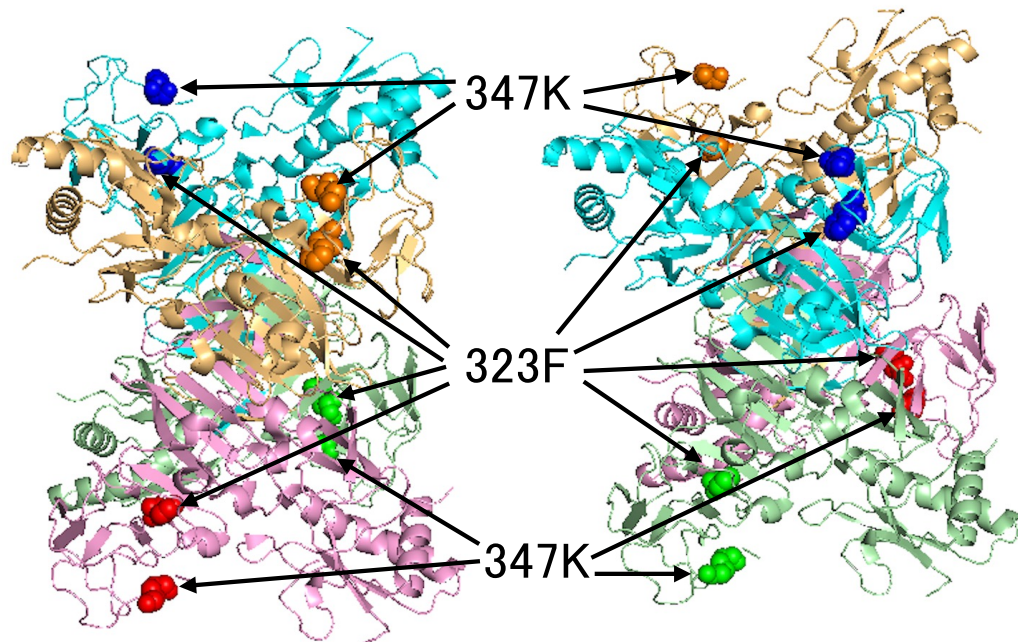

B

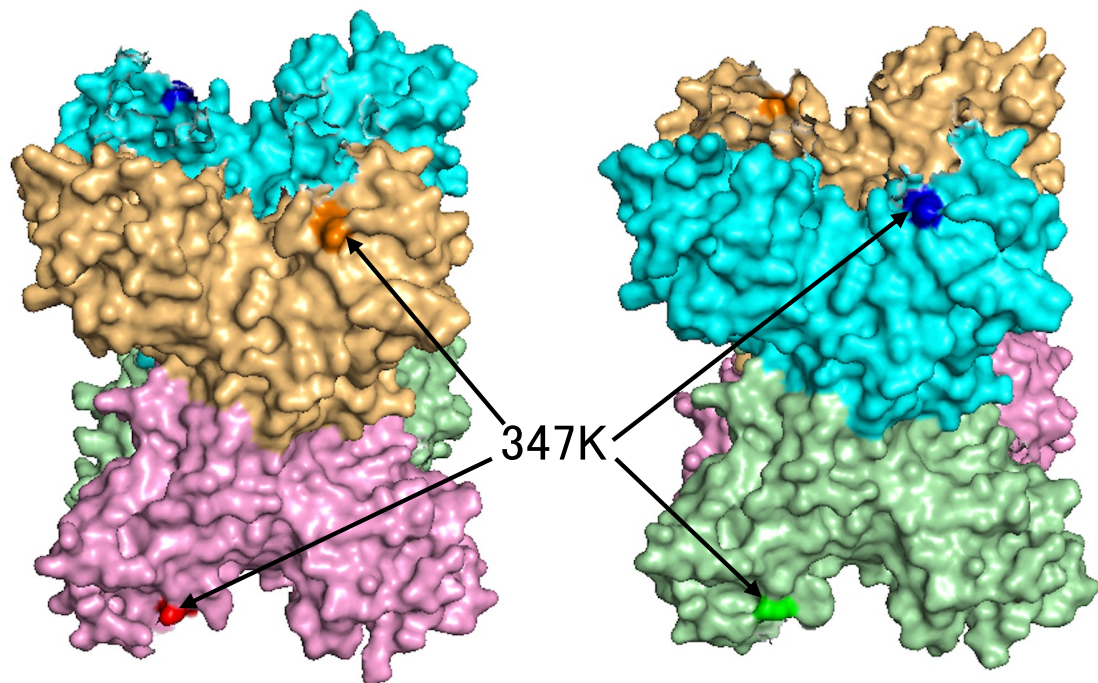

Supplement: Supplementary file 1 [file biosensors-15-00802-s001.zip › Figure S1.pdf]
